# Growth development of children and adolescents with inflammatory bowel disease in the period 2000–2014 based on data of the Saxon pediatric IBD registry: a population-based study

**DOI:** 10.1186/s12876-023-03088-5

**Published:** 2024-01-09

**Authors:** Xueming Zhou, Ivana Kern, Ulrike Rothe, Olaf Schoffer, Jens Weidner, Thomas Richter, Martin W. Laass, Joachim Kugler, Ulf Manuwald

**Affiliations:** 1 Health Sciences/Public Health, Institute and Policlinic for Occupational and Social Medicine, Faculty of Medicine “Carl Gustav Carus”, TU Dresden, 01309 Dresden, Germany; 2 GWT of the TU Dresden, Dresden, Germany; 3 Center for Evidence-Based Healthcare, University Hospital and Faculty of Medicine, Dresden, Germany; 4 Center for Medical Informatics, Institute for Medical Informatics and Biometry, Faculty of Medicine “Carl Gustav Carus”, TU Dresden, Dresden, Germany; 5 Clinic for Children and Adolescents, Hospital St. Georg, Leipzig, Germany; 6 Faculty of Medicine “Carl Gustav Carus”, University Hospital for Children and Adolescents, TU Dresden, Dresden, Germany; 7 University of Applied Sciences Dresden (FHD), Dresden, Germany

**Keywords:** Inflammatory bowel disease, Crohn’s disease, Ulcerative colitis, Growth failure, Growth disorders, Child, Height, Weight, Retardation

## Abstract

**Background:**

The incidence of inflammatory bowel disease (IBD) in children is on the increase worldwide. Growth disorders are common in pediatric patients with inflammatory bowel disease. The aim of this paper is to investigate anthropometric indicators, including height and weight in children with inflammatory bowel disease in Saxony, one of the German federal states, and to evaluate growth trends in patients by comparing their height and weight with that of healthy children in Germany.

**Methods:**

In Saxony, all children and adolescents with IBD were registered in the Saxon Pediatric IBD Registry from 2000 to 2014. The data used are therefore based on a total area-wide survey over 15 years. For this study, 421 datasets of children and adolescents aged 0–14 years with Crohn’s disease (CD) (*n* = 291) or ulcerative colitis (UC) (*n* = 130) were analyzed.

Z-score and percentile calculations were used to compare differences between IBD patients and the general population.

**Results:**

The children with CD or UC (both sexes) had a significant lower weight at diagnosis (the mean weight z-score had negative values) versus the general population. The weight values lay mostly below P50 (the 50th percentile, median), more precisely, mostly between P10 and P50 of the body weight child growth curve for corresponding sexes (KiGGS 2003–2006).

The height values of both sexes at diagnosis lay also mostly below P50 (the 50th percentile, median) of the child body growth curve for corresponding sexes (KiGGS 2003–2006), i.e. the mean height z-score was negative. But only the children with CD had a significant lower height, more precisely, mostly between P25 and P50 versus the general population (KIGGS). For children with UC the difference was not significant.

**Conclusion:**

In pediatric patients with IBD the possibility of growth disturbance, mainly in the form of weight retardation, is very probable.

## Background

Inflammatory bowel disease (IBD) is an idiopathic inflammatory disease of the intestine affecting the ileum, rectum and colon, which mainly includes Crohn’s disease (CD) and ulcerative colitis (UC). Linear growth disorders and delayed puberty are complications of childhood-onset IBD [1, 2]. The etiology of growth disturbances in IBD is multifactorial [3]. Malnutrition and the direct action of pro-inflammatory cytokines appear to be important explanatory factors [4].

The global incidence and prevalence of IBD has been increasing worldwide [5]. According to the German clinical practice guideline “Diagnosis and treatment of Crohn’s disease” (updated in 2014), the incidence of CD in Germany is up to 6.6 per 100,000 children and adolescents. The prevalence is approximately 100 to 200 per 100,000 children and adolescents [6]. For UC, the incidence in Germany is 3.0 to 3.9 per 100,000 children and adolescents, and the prevalence is about 160 to 250 per 100,000 children and adolescents [7]. Approximately 25% of all patients with inflammatory bowel disease (IBD) are diagnosed before the age of 18 years, with approximately one-quarter of all affected children and adolescents being under the age of 10 years at diagnosis [8]. For IBD in Saxony, the age-standardized incidence rates per 100,000 person-years increased from 4.6 [2.8; 6.3] in 2000 to 10.5 [7.5; 13.6] in 2009 [9].

Due to its chronic recurrent course, IBD endangers the physical, psychosocial and professional development of adolescents. Inflammatory bowel disease leads to a reduced quality of life in patients. Studying the disease patterns of children with inflammatory bowel disease provides evidence for improvements in health care delivery and the establishment of effective and efficient care structures [10].

Growth disturbances are more common in CD than in UC [3].

The aim of this paper is threefold. First, is to investigate anthropometric measures, including weight and height, of IBD patients at the time of diagnosis. Second, it is to determine whether patients diagnosed with inflammatory bowel disease in childhood have a lower body weight and height than the general population. Third, it is to determine whether the proportion of adolescents with childhood IBD who are below the median height and weight of the general population was less than 50%.

## Methods

### Data source

The Saxon Paediatric IBD Registry was established in 2000 to collect reliable and valid population-based epidemiological data and to observe trends in IBD in Saxony, Germany. All 31 children’s hospitals in Saxony provided data to the registry from 2000 to 2014. A total of 421 patients with IBD, who were under 15 years of age at onset and had anthropometric data were involved in the evaluation.

Data anlyzed of registered patients included date of birth, sex, type of diagnosis (CD or UC) and date of diagnosis. Anthropometric data included height and weight.

### Statistical analysis

The data were presented in the form of z-scores and percentile. Z-scores indicate how many standard deviations a certain value is away from the mean value of a data set. The value of the variable x (e.g. weight) can be converted to z-scores or standard deviation scores (SDS) using the formula z = ((x/M)^L^-1) / (S × L) for L ≠ 0 respectively z = 1/S × ln (x/M) for L = 0 [11]. A z-score < − 1 indicates a growth failure and a z-score < − 2 indicates short stature [12]. A percentile indicates the percentage of observations in a data set that had been below a certain value in the Study on the health of children and adolescents in German (KiGGS-Percentile) [11]. The null hypothesis that the percentage of children and adolescents with childhood IBD with a weight and height value below the median weight and height value of children in the general population equals to that of the general population was evaluated using a binomial test. Differences in the distribution of height and weight percentiles between CD and UC were evaluated using chi-square test. The level of significance was defined as α = 0.05. All statistical tests were performed using SPSS (IBM Corp., Version 22, Armonk, NY, USA) and Microsoft Excel Office Professional Plus (2010).

## Results

### The characteristics of the dataset

Four hundred twenty-one children under the age of 15 years at the time of IBD diagnosis had anthropometric data and were included. 250 of them were males (184 with CD and 66 with UC) and 171 females (107 with CD and 64 with UC). The median age at diagnosis was 12.0 years (range 0.8–15.0 years) in males and 12.3 years (range 0.6–15.0 years) in females.

### Weight

Body weight of CD children: The mean weight z-score for male children with CD at diagnosis was − 1.12 [95% CI -1.28; − 0.96]. 54.3% (*n* = 100) of them had a z-score < − 1 and 20.1% (*n* = 37) of them had a z-score < − 2. The mean weight z-score for female children with CD at diagnosis was − 1.01 [95% CI -1.22; − 0.80]. Of the female children with CD, 55.1% (*n* = 59) had a z-score of < − 1 and 20.6% (*n* = 22) had a z-score of < − 2.

Body weight of UC children: The mean weight z-score of male children at diagnosis was − 0.52 [95% CI -0.80; − 0.24]. 30.3% (*n* = 20) male children had a z-score < − 1, and 6.1% (*n* = 4) of them had a z-score < − 2. The mean weight z-score for female children at diagnosis was − 0.76 [95% CI -1.04; − 0.47]. 35.9% (*n* = 23) of the female children with UC had a weight z-score < − 1 and 15.6% (*n* = 10) had a weight z-score < − 2.

**Fig. 1:**
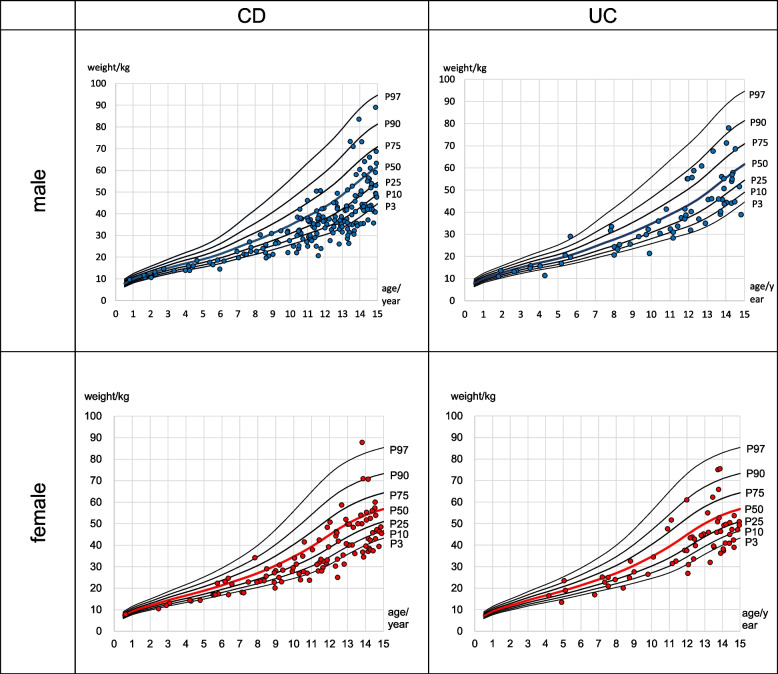
Weight of male and female children at diagnosis (CD and UC) plotted among the major percentiles of the general population (KiGGS)

Figure 1 shows the weight percentile of children under 15 years diagnosed with IBD in Saxony in relation to the main percentile curves for body weight (in kg) in male and female children (KiGGS 2003–2006) [11].

Weight at diagnosis was converted to a percentile and compared with the KiGGS percentile. 84.8% of male children with CD and 79.4% of female had a weight less than P50 (the KiGGS 50th percentile, median). 75.7% of male children with UC and 79.7% of female children were found to have a weight percentile < P50.

**Fig. 2:**
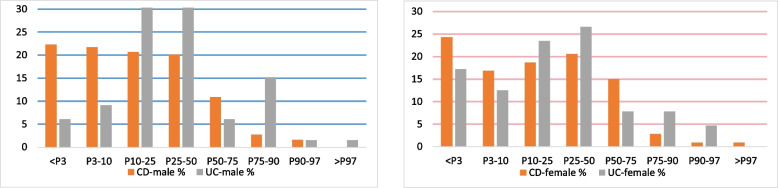
Distribution of weight percentile values (in %) of CD and UC between the major percentiles (KiGGS), separately for male and female

Figure 2 shows the weight of the children by sex and diagnosis grouped according to the major percentiles (KiGGS). On the Y-axis is shown the number of individuals as a percentage of the subgroup. It can be observed that there was a difference in the distribution of CD and UC in male and female children. The distribution curve for CD was more shifted to the left compared to that of UC. Both UC and CD were not symmetric around P50.

Table 1 summarizes the results of the statistical tests for weight percentiles among the different subgroups, as well as the comparative tests between the CD and UC groups. The results of the test show that the percentage of people under the weight of P50 is more than that of the general population. The distribution of weight percentiles in CD versus UC was significantly different only in male children and not in female children.

**Table 1:** A summary table of the results of statistical tests for weight (binomial test for probability percentage and chi-square for comparative test)

Diagnosis	Sex	Percentage	95%CI	*P* value
**Tests for probability percentage less than P50**				
**CD**	male	0.85	[0.79; 0.90]	*p* < 0.001*
	female	0.80	[0.72; 0.87]	*p* < 0.001*
**UC**	male	0.76	[0.64; 0.86]	*p* < 0.001*
	female	0.80	[0.68; 0.89]	*p* < 0.001*
**Comparative tests of the subgroups**				
**CD vs. UC**	male			*p* < 0.001*
	female			*p* = 0.226

*significant

### Height

Height of CD children: The mean height z-score for male children at diagnosis was −0.43 [95% CI -0.59; − 0.27]. 23.9% (*n* = 44) of male children had a z-score < − 1 and 9.8% (*n* = 18) had a z-score < − 2. The mean height z-score at diagnosis was − 0.40 [95% CI -0.61; − 0.18]. 30.8% (*n* = 33) of female pediatric patients had a height z-score < − 1 and 8.4% (*n* = 9) < − 2.

Height of UC children: There was no distinct difference in height compared to general population (mean height z-score at diagnosis for male children was − 0.10 [95% CI -0.36; 0.16], for female children the value was − 0.12 [95% CI -0.36; − 0.13]).

**Fig. 3:**
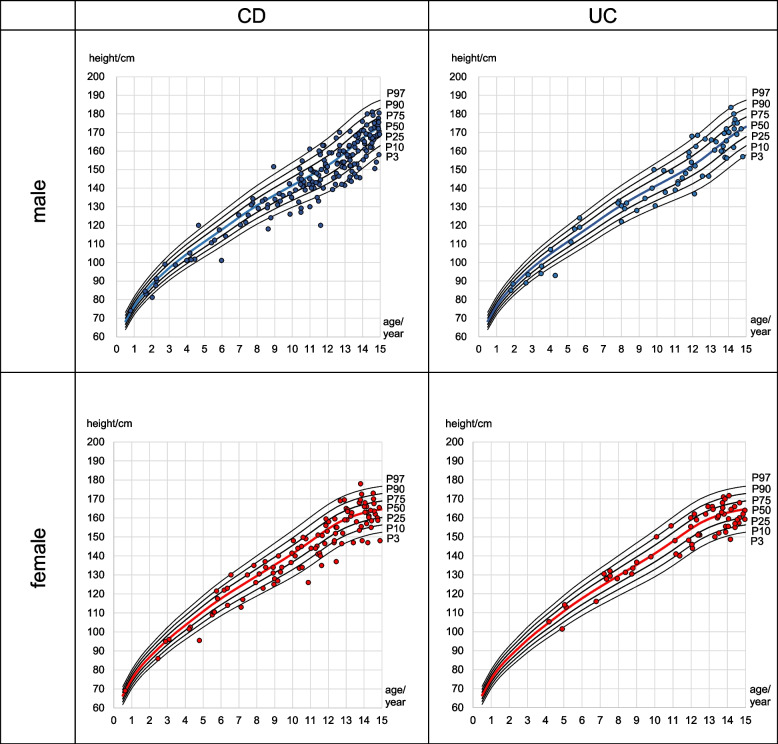
Height of male and female children at diagnosis (CD and UC) plotted among the major percentiles of the general population (KiGGS)

Figure 3 shows the height percentile of children under 15 years diagnosed with IBD in Saxony in relation to the main percentile curves for body height [cm] in male and female children (KiGGS 2003–2006) [11].

Height at diagnosis was converted to a percentile and compared with the KiGGS percentile. 67.4% of male children with CD and 62.6% of female had a height percentile less than P50 (the 50th percentile, median). When the height percentiles of UC children were compared with the KiGGS percentile, 51.5% of male children and 53.1% of female children were found to have a height percentile < P50.

**Fig. 4:**
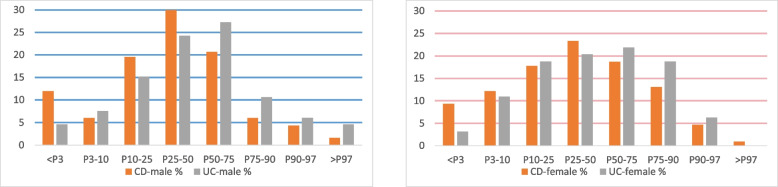
Distribution of height percentile values (in %) of CD and UC between the major percentiles (KiGGS), separately for male and female

Figure 4 illustrates the distribution of the children’s height between the major percentiles (KiGGS) separately by sex and diagnosis.

On the y-axis is shown the number of individuals as a percentage of the subgroup. It can be observed that the distribution of height percentiles is similar between CD and UC in both sexes.

Table 2 presents the results of the statistical tests for height percentiles among the different subgroups, as well as the comparative tests between the CD and UC groups. The results showed that in children with CD, the percentage of people with height less than P50 was slightly higher than that in the general population, but in children with UC there was no statistically significant difference. The distribution of height percentiles in CD versus UC was not significant.

**Table 2:** A summary table of the results of statistical tests for height (binomial test for probability percentage and chi-square for comparative test)

Diagnosis	Sex	Percentage	95%CI	*P* value
**Tests for probability percentage less than P50**				
**CD**	male	0.67	[0.60; 0.74]	*p* < 0.001*
	female	0.63	[0.53; 0.72]	*p* = 0.012*
**UC**	male	0.52	[0.39; 0.64]	*p* = 0.902
	female	0.53	[0.40; 0.66]	*p* = 0.708
**Comparative tests of the subgroups**				
**CD and UC**	male			*p* = 0.260
	female			*p* = 0.775

*significant

## Discussion

The main finding of this study was that weight retardation was apparent in patients diagnosed with inflammatory bowel disease in childhood, the percentage of CD children who are below P50 in weight and height is more than that of UC children. Inflammatory bowel disease in childhood is usually associated with weight and growth retardation [13] and is more common in patients with CD than in patients with UC [14], and more common in male patients than in female patients [14]. Most children already show growth disturbances at the time of diagnosis [15]. These results were also confirmed by this study.

There is controversy about the impact of a diagnosis of childhood IBD on final adult height [16]. A study of Mouratidou et al. (2020) has shown that patients with childhood onset IBD end up with an adult height only slightly lower than that of their healthy peers [17]. Amit et al. (2021) demonstrated that patients with CD were leaner, with a great proportion of subjects underweight [16]. Rinawi et al. (2020) state an increased proportion of pediatric onset IBD patients have growth impairment by adulthood. Male gender and diagnosis prior to puberty have been identified as risk factors for reduced adult height in both diseases [18]. In this work, development of patients could be observed until the age of 18 years, with 31.1% paediatric patients reaching the median standard weight and 40.5% reaching the median standard height at age 18 of the child growth curve.

The causes of growth disturbances in children with IBD are multifaceted and poorly understood [3]. Inflammation, malnutrition and steroid treatment are the main determinants [19]. Rinawi et al. (2020) proposed parental height has a significant impact on the adult height of children with CD [18]. Diagnostic tools and treatment options may also have an impact on the growth of children with IBD. Accurate diagnosis of IBD can improve growth outcomes by reducing the delay in diagnosis and selecting appropriate treatment options as early as possible. The establishment of our database has contributed to the creation and improvement of an effective and efficient care structure for IBD health services. Through the in-depth analysis of the patient cohort with IBD: disease incidence, disease severity, disease progression, trends, but also through the body weight and height analysis described in this article, the Saxon pediatric IBD Registry provides a scientific basis for informed healthcare decisions. Specifically, more attention should be paid to the nutrition of pediatric IBD patients, e.g. by specially trained nutritionists, so that the weight and height retardation of children could be reduced.

## Strengths and limitation

The Saxon pediatric IBD Registry - our database provides information on 96.7% of pediatric patients with IBD in Saxony, Germany from 2000 to 2014. The database is relatively complete and includes anthropometric indicators present in 71.4% of patients at the time of diagnosis. We included anthropometric data for each child at the time of diagnosis (anthropometric measurements dated within 45 days of the date of diagnosis) to avoid the effect of treatment on weight and height. This information is important for understanding trends in this disease and developing new treatments.

The results of the analysis in this study also have some limitations. The sample size is not very large, the evaluation is based on data collected 15 years nationwide in the whole of Saxony. The data did not cover all age groups from 0 to 15 years without gaps, especially in children with very early onset.

## Conclusion

Growth disorders are a complication of childhood inflammatory bowel disease, especially in patients with CD. In IBD patients younger than 15 years of age, growth disturbances, especially in the form of weight retardation, are likely.

More than three quarters of children with CD and UC have weight values below the P50 of the child growth curve. About two thirds of children with CD and about one half of children with UC have height values below P50 of the child growth curve. Over 50 % of children with CD and over a quarter of children with UC had a weight percentile less than P16 (z-score < − 1) and approximately a quarter of children with CD and a fifth of children with UC had a height percentile less than P16 (z-score < − 1). The percentage of children with IBD who weigh less than P50 regarding the general population is greater than 50%. The percentage for the number of CD children below P50 is slightly greater than 50%, but there is no statistical difference between children with UC and the general population.

## Data Availability

All data generated or analyzed during this study are included in this published article.

## Abbreviations

CD: Crohn’s disease
CI: Confidence interval
cm: centimeter
IBD: Inflammatory bowel disease
kg: kilogram
KiGGS: “German health interview and examination survey for children and adolescents”
L: Incline
M: Median
P50: Percentile 50
S: Coefficient of variation
SDS: Standard deviation scores
UC: Ulcerative colitis
